# Evaluation of genetic diversity and selection signals in gayal populations across four countries through whole-genome resequencing

**DOI:** 10.1007/s44154-025-00252-7

**Published:** 2025-10-26

**Authors:** Xin Liu, Qiaoxian Li, Jianyong Liu, Zulfiqar Ahmed, Jicai Zhang, Zhe Wang, Ankui Wang, Ningbo Chen, Yongzhen Huang, Gang Ren, Hong Chen, Chuzhao Lei, Bizhi Huang

**Affiliations:** 1 Yunnan Academy of Grassland and Animal Science, Kunming, 652100 China; 2 Key Laboratory of Animal Genetics, Breeding and Reproduction of Shaanxi Province, College of Animal Science and Technology, Northwest A&F University, Yangling, 712100 China; 3 Department of Livestock and Poultry Production, Faculty of Veterinary and Animal Sciences, University of Poonch Rawalakot, Rawalakot, 12350 Pakistan

**Keywords:** Gayal, Genetic diversity, Mitochondrial genome, Selection signal, Whole-genome resequencing

## Abstract

Gayal (*Bos frontalis*) an endangered bovine species inhabitingChina, India, Bangladesh, Myanmar and Bhutan, has a mysterious evolutionary origin. Shaped by natural selection, its unique traits make it a valuable genetic resource; however, its populations are rapidly declining. In this study, comprehensive whole-genome resequencing of fifty-eight samples of Gayal from China, India, Myanmar and Bangladesh was performed. We identified over 44 million SNPs across four Gayal populations. Nucleotide diversity analysis revealed variations in genetic diversity, with the lowest occurring in India and the highest occurring in China. Phylogenetic tree analysis revealed three distinct clades representing China, India and Bangladesh-Myanmar, which were further confirmed by principal component and admixture analyses. The genetic exchanges between Gayal and other bovine species indicate limited influence from domestic cattle in both the Chinese and Bangladeshi Gayal populations. Mitochondrial DNA sequences and a phylogenetic tree highlighted the unique mitochondrial genome of Gayal. Genome-wide selection signals pinpointed candidate genes linked to mitochondrial function, immunity, musculoskeletal development, reproduction and growth performance. Distinct haplotype patterns emerged for the *CCDC157*, *KIAA0753* and *MTFP1* genes in the Chinese and Bangladesh-Myanmar Gayal populations, indicating artificial selection in the Chinese population. KEGG pathway and gene ontology enrichment analyses provided insights into processes related to neurodevelopment, cardiac function, tissue growth, immunity and metabolism. In summary, our study enhances our understanding of Gayal genetics, population structure and selection signals across four countries. This knowledge is crucial for conserving this endangered species amid its rapid decline.

**Supplementary Information:**

The online version contains supplementary material available at 10.1007/s44154-025-00252-7.

## Introduction

Gayal, commonly known as mithan, represents a distinctive semiwild and critically endangered bovine species that primarily inhabits hilly regions across China, India, Bangladesh, Myanmar and Bhutan (Mondal et al. 2004). The exact origin of Gayal has remained elusive, with divergent hypotheses such as that it is an autonomous species (Baig et al. 2013); Ma et al. 2007); Shan et al. 1980) or a hybrid descendant of crossbred wild Gaur and domestic cattle (Gou et al. 2010); Lan et al. 1993). To validate the hypotheses about Gayal origin, a detailed investigation of the Gayal evolutionary history and ancestral fragment is needed. Interestingly, Gayal can interbreed with indicine and taurine cattle, yielding fertile female offspring; however, the fertility of males is questionable (Giasuddin et al. 2003; Huque et al. 2001; Nyunt and Win 2004).

Phenotypically, Gayal has distinctive features due to the influence of natural selection, which differentiates it from domestic cattle. A prominent forehead ridge, short and broad ears and distinctive white stockings over feet are salient phenotypic traits of Gayal (He et al. 2009; Qu et al. 2012). In addition, Gayal is distinguished from *Bos indicus* (60 chromosomes), *Bos taurus* (60 chromosomes) and *Bos gaurus* (56 chromosomes) due to its unique chromosomal configuration (58 chromosomes) (Chi et al. 2004; Qu et al. 2012; Xi et al. 2012).

Gayal consumes a wide range of flora, from tree and bamboo leaves to grasses and weeds, with a fondness for salt (Deng et al. 2007; Uzzaman et al. 2014). Gayal has superior body conformation and meat quality with prime tenderness attributes compared to indigenous domestic cattle, making it a valuable genetic source for cross-breeding with domestic cattle to acquire improved meat and milk traits from their hybrids (Ge et al. 1996; Giasuddin et al. 2003).

The origin of the Gayal has always been a matter of debate. Currently, there are mainly the following three theories: First theory is Gayal are domesticated from the Gaur. Chi et al. (2004) analyzed the chromosomes of a male Gayal fromChina and found that there was a chromosomal fusion in the Gayal, corresponding to the 2 nd and 28th chromosomes of domestic cattle. The same chromosomal fusion has also been reported in Gaur (Gallagher and Womack 1992), thus they support the conclusion that Gayal were domesticated from Gaur. Second theory is Gayal are a hybrid of Gaur and domestic cattle. (Mei et al. 2016) constructed a phylogenetic tree using the assembled mitochondrial DNA data of Gayal and the downloaded mitochondrial DNA data of other closely related cattle species. The results indicated that, in the evolutionary tree constructed from the mitochondrial genome, the Gayal are most closely related to Taurus. Third theory is Gayal are originated from a close relative species of Gaur that may be extinct now. (Wu et al. 2018) performed a population history simulation analysis on a Gayal and Gaur and the results indicated that the Gayal and Gaur may have begun to diverge as early as 994,000 years ago, thus suggesting that it is not a domesticated breed of Gaur. The difficulty in collecting samples of Gayal has led to most studies being based on the analysis of just one or a few individuals, resulting in inconsistent conclusions. Thus, further in-depth research and discussion are still needed to elucidate this issue.

Despite its aforementioned significance, Gayal populations are rapidly declining and are therefore classified as endangered species by the International Union for Conservation of Nature and Natural Resources (IUCN). There is limited literature available on Gayal genetics, which has focused on meat performance (Uzzaman et al. 2014), mitochondrial DNA genetic diversity (Gou et al. 2010) and the construction of genome assembly (Mei et al. 2016). However, systematic studies on the Gayal genome are direly needed, especially with larger datasets. Recognizing this critical research gap, our study encompasses fifty-eight whole-genome sequences of Gayal from a wide geographical area of China, India, Myanmar and Bangladesh. The objective of this study was to gain a comprehensive understanding of genetic intricacies and deepen the body of knowledge about the evolutionary genetics of Gayal through the use of selection signature detection methods.

## Results and discussions

### Sequencing read depth statistics and SNP identification

We obtained 2 billion reads with an average alignment length of 99.74% and a coverage range from 4.54 × to 20.96 × with an average depth of 8.15 ×. The identified single nucleotide polymorphism (SNP) in the dataset of 58 samples amounted to 15 Gb of the reference genome, over 44 Mbs SNPs with a Ts/Tv value of 2.52. The majority of the annotated SNPs were identified in intergenic and intron regions.

### Population genetic diversity and relationships

**Fig. 1:**
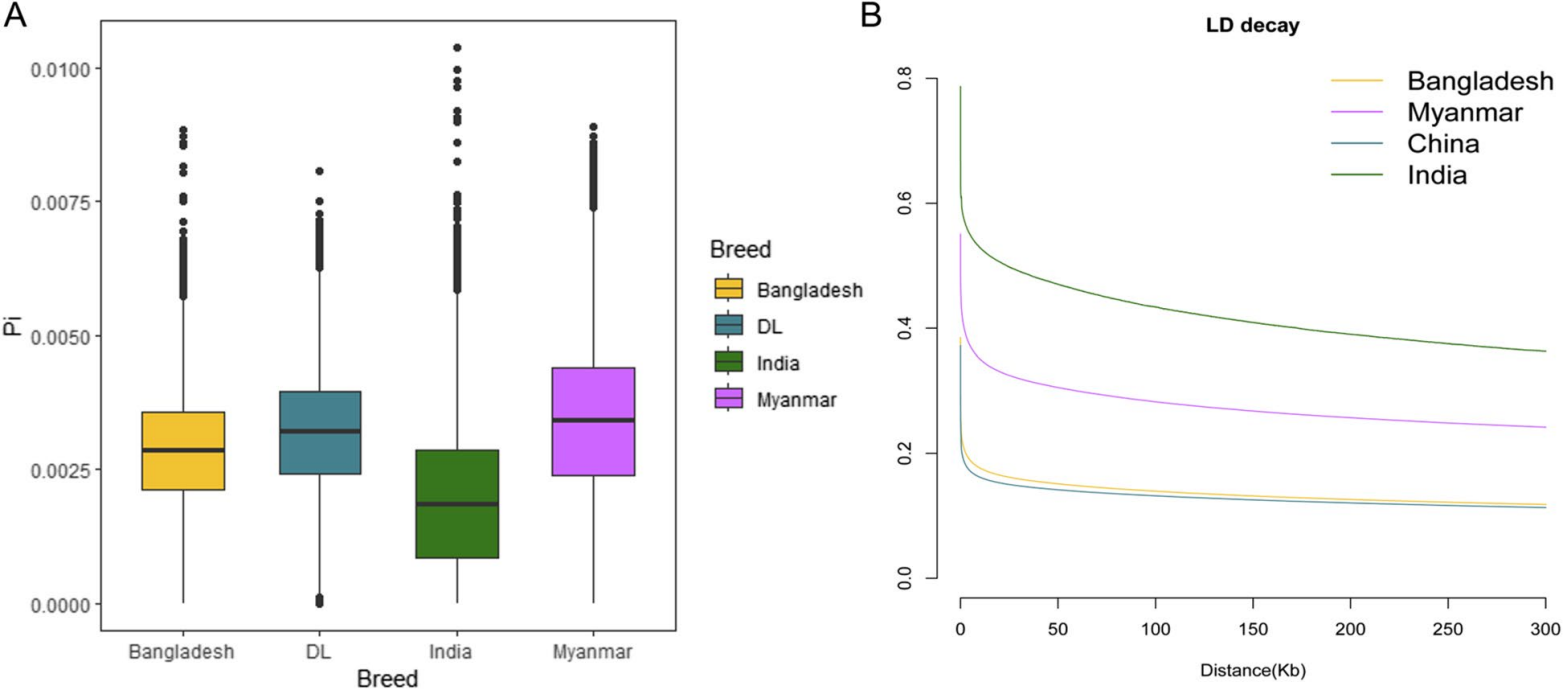
Summary of genomic variation statistics of the four Gayal groups. **A**Genome-wide nucleotide diversity distribution of each group in 50 kb windows with 50 kb increments. The horizontal line inside the box shows the median of the distribution. The box boundaries represent the first and third quartiles and the points represent outliers. The outliers are the data points that fall outside the whiskers. **B** Estimated genome-wide average LD decay from each group

The computed nucleotide diversity across different Gayal populations revealed the following pattern: Indian < Bangladesh < Chinese < Myanmar Gayal (Fig. 1A). The results of linkage disequilibrium (LD) analysis indicated that LD decay was the slowest in Indian Gayal, followed by Myanmar and Bangladesh Gayal. In contrast, Chinese Gayal exhibited the fastest LD decay, signifying that the Chinese Gayal population possesses the highest genetic diversity (Fig. 1B).

It can be seen that the genetic diversity of Indian gayal is the lowest, while that of Chinese gayal is the highest. However, this result seems counter intuitive when considering the current population statistics of gayal (Dorji et al. 2021). Since the data for these three Indian gayal was downloaded from public databases, we speculated that this might be due to kinship relationships among them.

### Population genetic structure and phylogenetic analysis

**Fig. 2:**
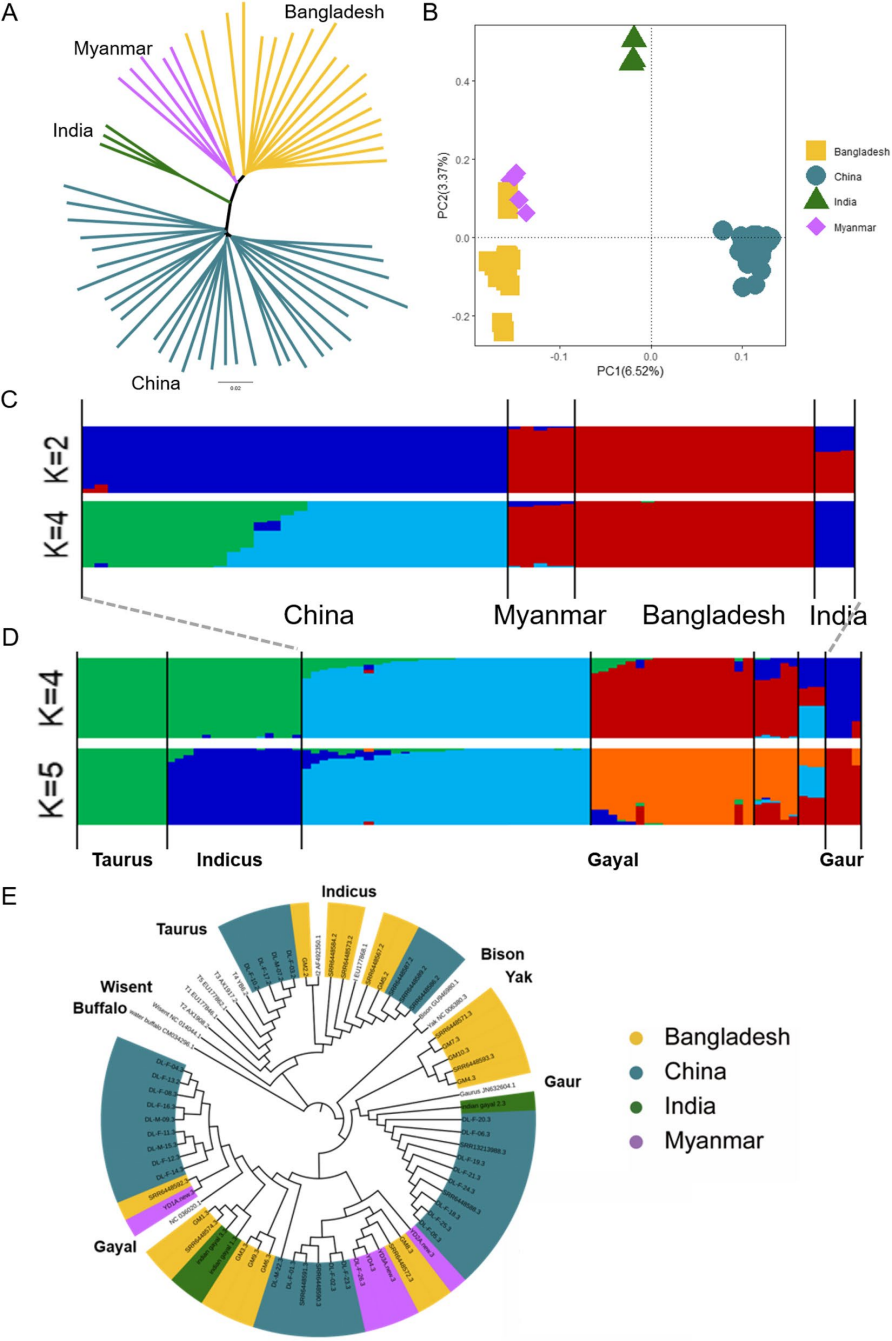
Population structure and relationships of Gayal (**A**) Neighbor-joining phylogenetic tree constructed using whole-genome SNP data. **B** The principal component analysis (PCA) plot shows PC1 versus PC2. **C** Model-based clustering of Gayal using ADMIXTURE with *K* = 2, 4. The different colors reflect the different cattle ancestry. **D** Model-based clustering of Gayal, Taurus, Indicus and Gaur using ADMIXTURE with K = 4, 5. The different colors reflect the different cattle ancestry. **E** Mitochondrial phylogenetic tree of Gayal. Individuals without background color are known haplotype download sequences

The neighbor-joining tree (NJ tree) results demonstrated that the Gayal population is primarily categorized into three clades: Chinese Gayal forms one clade and Bangladesh and Myanmar form a closer cluster (Fig. 2A). In contrast, Indian Gayal falls in a separate clade. These results highlight distinct regional distribution patterns and genetic differences between the Chinese Gayal and Southeast Asian Gayal population. Additionally, the principal component analysis (PCA) results revealed that Gayal from the Chinese region formed a distinct cluster and Myanmar and Bangladesh were in closer proximity (Fig. 2B). This observation aligns with the results of the NJ tree and further supports the phylogenetic patterns. When applying the ADMIXTURE analysis with *K* = 2, two primary groups emerged: the Chinese region and the Bangladesh-Myanmar region, Gayal. When *K* = 4, the Chinese regional Gayal group is further subdivided into two ancestral lineage components (Fig. 2C). However, Southeast Asian Gayal populations maintained their division between Bangladesh-Myanmar and Indian lineages.

Through NJ tree, PCA and ADMIXTURE analysis results, it can be observed that there is a certain degree of differentiation among the internal populations of the Gayal. The Gayal in China shows a significant genetic difference from the Gayal in Southeast Asia, particularly from the Gayal in the Bangladesh-Myanmar region. The possible reasons for this phenomenon may include the following: (1) Geographical isolation caused by natural factors such as mountains and rivers may have hindered gene flow between different Gayal populations, leading to geographical isolation from the Gayal in Southeast Asia over a long period of natural selection. (2) The Gayal in China is mainly herded by the Dulong ethnic group. There may be human factors that could subtly influence the Gayal population in China. This may affect the traits of the population such as morphology, growth and development, immunity and reproduction.

### Genetic exchange of Gayal with other bovine species

The autosomal SNP data of 58 Gayal individuals was analyzed alongside data from three closely related cattle varieties to determine ancestral fragments (Fig. 2D). At *K* = 4, a slight genetic influence from domestic cattle was evident in both the Chinese and Bangladesh Gayal populations. However, at *K* = 5, a distinct separation between the Taurus and Indicus lineages became apparent. The Chinese group displayed a minor influence of *Taurus* in addition to the influence of *Indicu*s, while the Bangladesh group predominantly exhibited the influence of *Indicus*.

The maximum likelihood tree (ML tree) results revealed four Chinese Gayal individuals clustered with the *Taurus* T4 clade (YB6.2), five Bangladesh individuals and three Chinese Gayal individuals clustered with the *Indicus* clade (1 Bangladesh Gayal belonged to clade I2; I2 AF492350.1) and the rest belonged to clade I1 (I EU177868.1) (Fig. 2E). One clade (30 individuals) clustered with Gayal (NC036020.1) and the other (16 individuals) clustered with Gaur (Gaurus JN632604.1); however, none were found in the cluster of American bison, European bison, or yak haplotypes.

Based on the analysis of genetic structure and mitochondrial DNA, we believe that the ancestors of the Gayal are more likely to be close relatives of the Gaur, rather than the other two hypotheses, which is consistent with the perspective of Wu et al. (Wu et al. 2018). In the ML tree result, mitochondrial haplotypes of domestic cattle were detected in the Gayal population, which is in agreement with the findings of previous studies (Gou et al. 2010; Tanaka et al. 2011). Four Chinese Gayal clusters in the*Taurus*T4 clade, which is haplotype specific to East Asian cattle and is distributed mainly in Eastern Siberia, China, Korea and Japan (Mannen et al. 2004). The presence of domestic cattle mitochondrial haplotypes in the Gayal population suggested genomic infiltration, indicating artificial introduction during domestication. To maintain a pure Gayal lineage in future breeding and expansion, removing individuals with domestic cattle ancestry is worth considering (Supplementary Table S4). The rest Gayal individuals shows a close relationship between the clades represented by Gaur and Gayal and the clustering of these clades in the tree diagram structure is indicative of common ancestry and close genetic affinity.

### Selection signals for the Chinese Gayal and Bangladesh-Myanmar Gayal

**Fig. 3:**
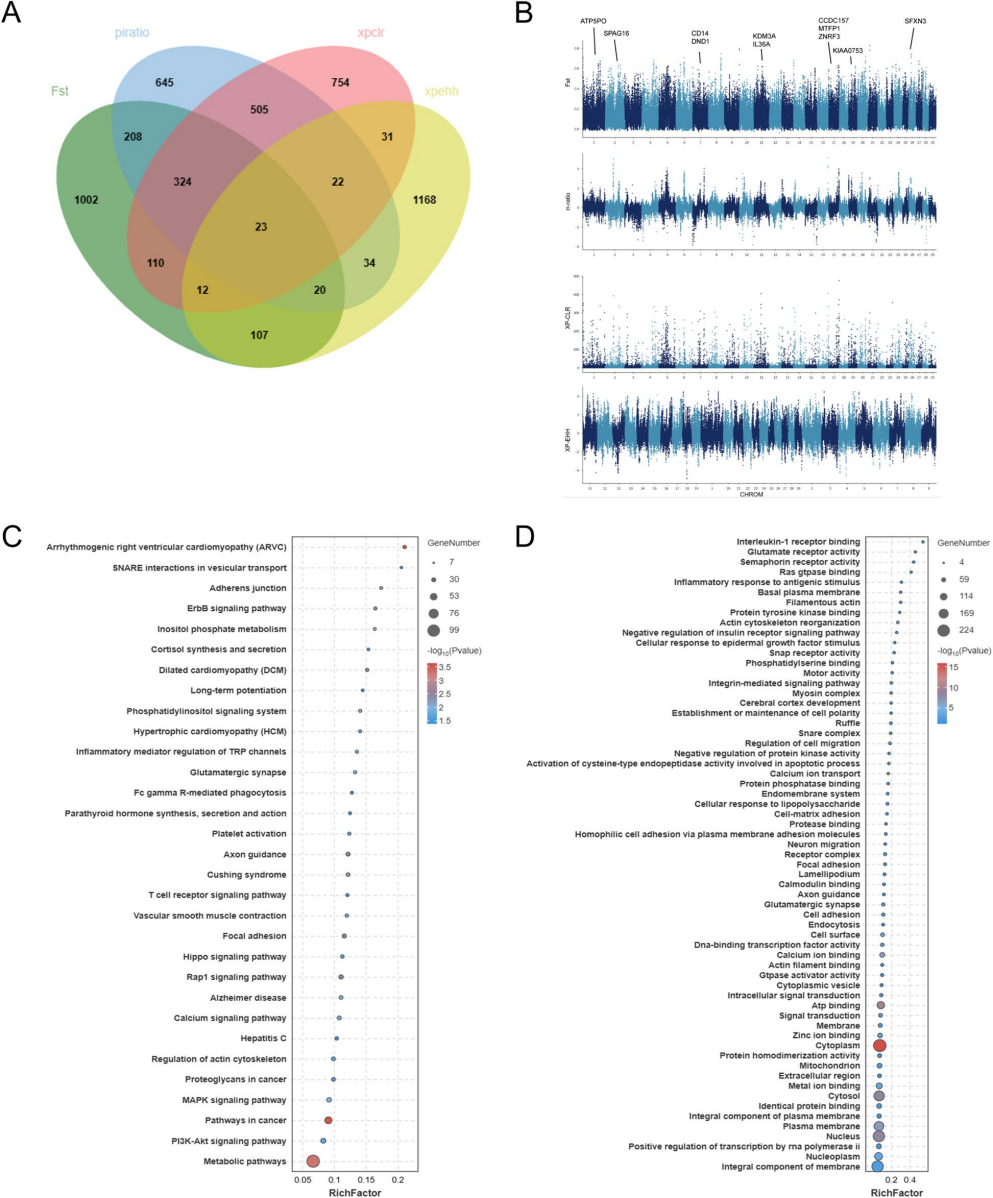
Selection signals in the Chinese Gayal and Bangladesh-Myanmar Gayal genomic areas. **A** Overlapping results of the four methods. **B** The Manhattan plot of selected sweeps for the Chinese Gayal and Bangladesh-Myanmar Gayal cohorts (*F*_ST_, π-ratio, XP-EHH and XP-CLR). **C** KEGG pathway enrichment analysis of candidate genes. **D** Gene Ontology (GO) enrichment analysis of candidate genes

We used theChinese regional Gayal as target population and the Bangladesh-Myanmar Gayal as reference population. From the top 5 % of the regions, the genes identified by at least two methods were regarded as candidate genes (Supplementary Table S5-S8). The results of the selection signals, as identified by four distinct methodologies, fixation index (*F*_ST_), nucleotide diversity ratio (θπ-ratio), cross population composite likelihood ratio (XP-CLR) and cross-population extended haplotype homozygosity (XP-EHH), are presented by Venn diagram (Fig. 3A).

We identified several key genes (*ATP5PO*, *SFXN3* and *KDM3A*) that play crucial roles in regulating mitochondrial and cardiovascular functions. *ATP5PO* is essential for oxidative phosphorylation and is responsible for generating ATP. Mutations in *ATP5PO *can lead to defective complex V assembly, contributing to Leigh syndrome (Ganapathi et al. 2022). The *SFXN1 *gene functions as a mitochondrial serine transporter in one-carbon metabolism (Kory et al. 2018). *KDM3A *plays a critical role in regulating mitochondrial biogenesis by sensing oxygen levels and controlling PGC-1α (Qian et al. 2019).

Additionally, the annotated genes were also associated with a variety of vital aspects of life, such as immunity (*CD14* and *IL36A*) (Patrick et al*.* 2021); (Wu et al. 2019), reproduction (C*CDC157*, *DND1 and SPAG16*) (Yamaji et al. 2017; Yuan et al. 2019; Zhang et al. 2009), growth performance (*KIAA0753*) (Hammarsjo et al. 2017) and lipid metabolism (*MTFP1* and *ZNRF3*) (Fig. 3B) (Belenguer et al. 2022; Patitucci et al. 2023).

The results of the Kyoto Encyclopedia of Genes and Genomes (KEGG) and Gene Ontology (GO) enrichment analyses of the top genes revealed the significant enrichment of these genes in pathways related to neurological development, cardiac function, tissue growth, immunity and metabolism (corrected *P* value < 0.05) (Fig. 3C-D). Notably, pathways such as metabolic, arrhythmogenic right ventricular cardiomyopathy (ARVC) and vascular smooth muscle contraction were enriched. Additionally, GO analysis revealed associations with immune responses, neurological processes and actin-related aspects (corrected *P* value < 0.05). These pathways included inflammatory responses, interleukin-1 receptor binding, axon guidance and glutamatergic synapses (Supplementary Table S9 and S10).

**Fig. 4:**
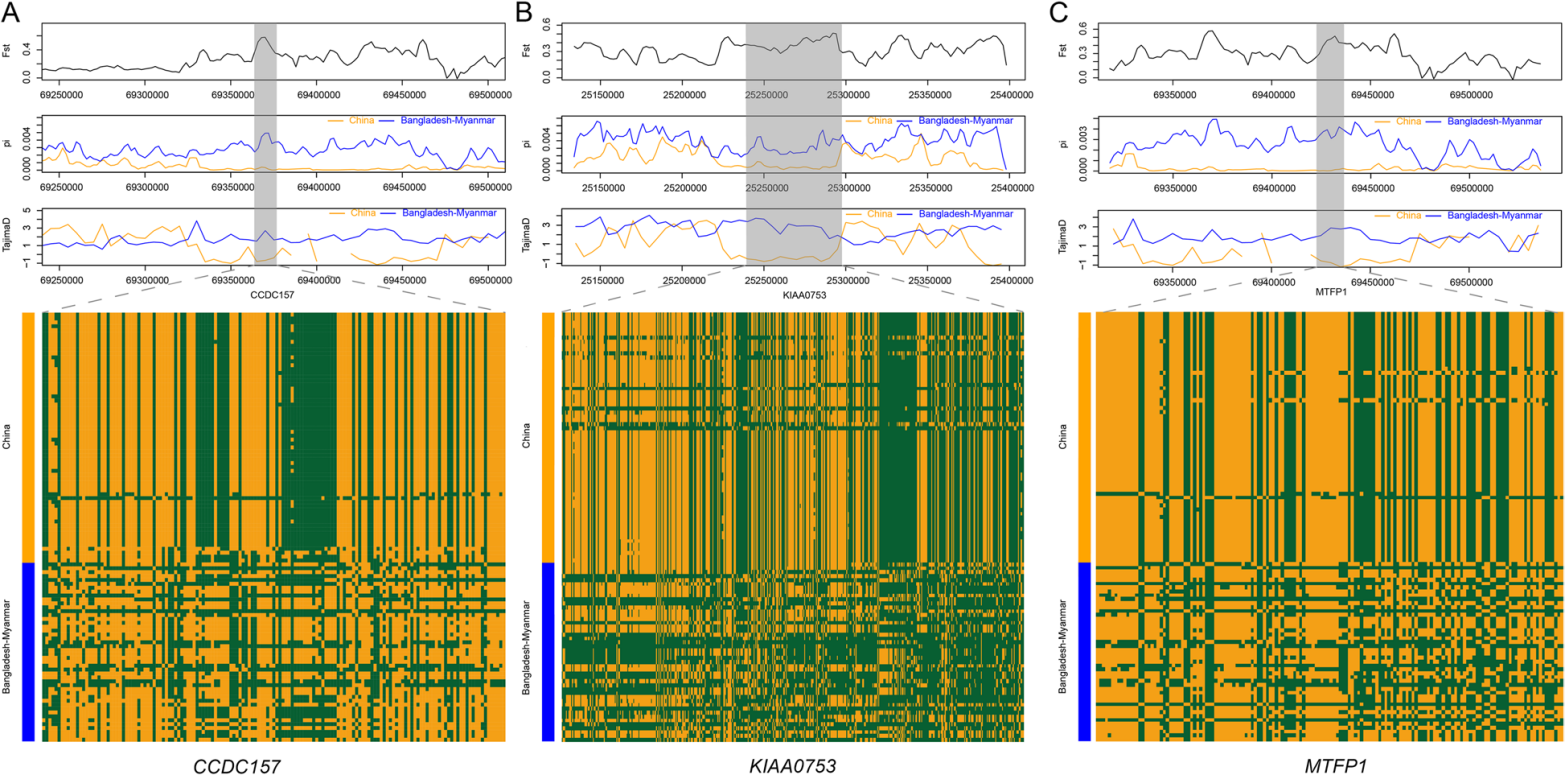
Haplotype patterns and line charts of the *F*_ST_, π and Tajima D values of the three genes. **A** Haplotype of the *CCDC157* gene. **B** Haplotype of the *KIAA0753* gene. **C** Haplotype of the *MTFP1* gene

Furthermore, haplotype tests for specific candidate genes (*CCDC157*, *KIAA0753* and *MTFP1*) were used to assess genetic diversity and haplotype patterns in the Chinese and Bangladesh-Myanmar populations (Fig. 4A-C). These tests revealed distinct haplotype patterns and lower genetic diversity in the Chinese Gayal population.

These three genes may have significant economic value for cattle breed selection. *CCDC157*is likely related to reproductive traits. This gene is downregulated in men with idiopathic non-obstructive azoospermia (NOA) and its homolog in male fruit flies leads to male sterility when absent (Yuan et al. 2019). The*KIAA0753*gene is possibly related to growth and development. Its mutations can cause developmental delay in humans or rib dysplasia (Hammarsjo et al. 2017; Stephen et al. 2017). The*MTFP1*gene has been confirmed to be related to fat production (Patitucci et al. 2023). And these three genes share the same haplotype patterns, which are relatively consistent in the Chinese Gayal population, while in the Bangladesh-Myanmar gayal population, the SNP haplotypes are relatively chaotic. This indicates that the Chinese gayal population may have been under selective pressure. Considering that the functions of these three genes are mostly related to the economic traits of cattle, we speculate that this selective pressure is likely due to artificial selection. Due to the geographical characteristics of Yunnan, it has limited gene flow with the Bangladesh-Myanmar gayal. As a result, this haplotype pattern has been fixed.

## Conclusion

Using whole-genome resequencing analysis Gayal and other other bovine species, we believed that Gayal is most likely to be close relatives of the Gaur, rather than domesticated from the Gaur or hybrid of Gaur and domestic cattle. Genetic exchanges with domestic cattle were limited, emphasizing the need to protect Gayal's unique genetics. we also found that Gayal genetic differences in Gayal populations from China, India, Myanmar and Bangladesh were explored. Different Gayal population clusters are aligned with different geographical regions. In addition, used Chinese gayal as the target population for selective analysis, candidate genes linked to critical functions, such as mitochondrial dynamics, immunity, musculoskeletal development, reproduction and growth, were identified in the Gayal population. Distinct gene patterns in the Chinese and Bangladesh-Myanmar Gayal populations suggested artificial selection in China. But further research on the Indian gayal awaits more sequencing data from a larger sample size for in-depth analysis.

## Materials and methods

### Sample collection, DNA extraction and Illumina sequencing

In this study, we collected ear tissue samples from unrelated Gayal (*n* = 36), including Chinese (*n* = 25), Bangladesh (*n* = 10) and Myanmar (*n* = 1) Gayal.

DNA extraction was performed using the phenol–chloroform method (Sambrook 1989), with detailed operations as follows:

First, a small piece of ear tissue was cut and finely ground, treated with lysis buffer, then an equal volume of phenol–chloroform mixture was added. After thorough mixing, the mixture was centrifuged, the supernatant was taken and 75% ethanol was added to wash away impurities. This process was repeated twice and after drying, a small amount of double-distilled water was added to dissolve the DNA precipitate, which was then used for subsequent concentration determination. Illumina paired-end libraries with an insert size of 350 bp were constructed using the NEB Next® Ultra™ DNA Library Prep Kit for Illumina (NEB, USA) following the manufacturer’s recommendations. The Illumina paired-end was sequenced on the Illumina platforms and 150 bp paired-end reads were generated.

Obtained clean reads files for subsequent analysis. After thorough analysis of each sample paired-end libraries were assembled with an average insert size of 300 base pairs (bp) and an average read length of 150 bp. Sequencing was performed using the Illumina NovaSeq platform and the sequencing process was performed by an expertly handling the Beijing NoHo sequencing company. Additionally the published data of 22 Gayal samples were downloaded from the National Center for Biotechnology Information (NCBI)(7 fromChina, 8 from Bangladesh, 3 from India and 4 from Myanmar Gayal). A whole-genome dataset of 58 samples was constructed and utilized for subsequent processing. Detailed information about the samples and geographic origin of the breeds used is presented in Supplementary Table S1.

### Mapping and SNP calling

The genotype data were generated by following the 1000Bull Genomes Project Run 8 guidelines. We used Trimmomatic v.0.39 (Bolger et al. 2014) to remove low-quality bases and artifact sequences. The clean reads were mapped to the bovine reference assembly (ARS-UCD1.2) using BWA-MEM v.0.7.13-r1126 (Bolger et al. 2014). SAMtools v.1.9 (Li et al. 2009) was used to sort the Bam files and Picard v.2.20.2 (http://broadinstitute.github.io/picard) was used to identify potential Polymerase Chain Reaction (PCR) duplicates for the mapped reads. Base quality score recalibration (BQSR) was performed on the mapped reads using the Genome Analysis Toolkit (GATK, v.3.8–1-0-gf15c1c1ef) and a known variant file from the 1000 Bull Genomes Project (McKenna et al. 2010). The GVCF files were generated with GATK’s ‘HaplotypeCaller’ for SNP calling and with ‘GenotypeGVCFs’ and ‘SelectVariants’ selected candidate SNPs from combined GVCF files. VariantFiltration in GATK was used to identify SNP clusters and filtering criteria were applied. The SNP filtering criteria included a mean depth of 1/3, quality by depth (QD) > 3, variant quality score (QUAL) > 30, strand odds ratio (SOR) > 3, Fisher strand (FS) > 60, mapping quality (MQ) > 40, mapping quality rank sum test (MQRankSum) > 12.5 and read position rank sum test (ReadPosRankSum) > 8. Nonbiallelic SNPs and SNPs with missing genotype rates > 0.1 were removed. BEAGLE v.4.0 simultaneously imputed and phased SNPs, filtering those with DR2 < 0.05 (Browning and Browning 2007). The remaining SNPs were annotated using ANNOVAR based on their positions (Wang et al. 2010).

### Genomic diversity analysis

Nucleotide diversity was assessed through the sliding window method using VCFtools v4.1 software (Danecek et al. 2011). The genome was divided into 50 kb windows, incrementally stepping by 20 kb along the genomic sequence. The LD and *r*_2 _ statistics were computed using PopLDdecay software with default parameters (Zhang et al. 2019), taking into account the physical distance between pairwise SNPs because of the physical distance between pairwise SNPs and default parameters. To illustrate the breakdown of LD at increasing distances from the central core allele, a haplotype bifurcation diagram was generated using the R package rehh v.3.01 (Gautier M et al. 2017) based on phased haplotypes obtained with Beagle v.4.1 (Browning and Browning 2007).

### Population genetic structure

We constructed a phylogenetic tree from the 58 samples using PLINK (Purcell et al. 2007)and MEGA v11.0. The tree was visualized and refined using iTOL (Kumar et al. 2016)and then visualized and subjected to beautification with iTOL (Letunic and Bork 2016). For PCA and admixture analysis, we filtered out SNPs with a minor allele frequency (MAF) < 0.01 and performed LD-based pruning for the genotype data using the –indep-pairwise 50 10 0.1 option of PLINK v1.9. Then we used the Smartpca program in EIGENSOFT v4.2 for PCA analysis (Patterson et al. 2006). The ADMIIXTURE v1.3.0 (Alexander et al. 2009) was used to quantify genome-wide admixture among cattle populations and run for each possible group number (K = 2–5), where K was the assumed number of ancestries.

### Analysis of the genetic exchange of Gayal with bovine species

We extracted complete mitochondrial DNA sequences from the BAM files of 58 Gayal samples using SAMtools and converted these sequences into FASTQ format using the SamToFastq module of Picard software. The mitochondrial genomes of these samples were assembled with Mapping Iterative Assembler (MIA) software utilizing specific parameters (H 1-F-i-c-r) as previously described (Briggs et al. 2009). To enhance the rigor of our dataset and analysis, we combined the FASTQ files with mitochondrial genomes from closely related cattle species obtained from NCBI (Supplementary Table S2 and S3). We constructed a maximum likelihood tree using IQtree (Nguyen et al. 2015) to check the shared genomic regions and visualized them alongside the mitochondrial genomes using iTOL (Letunic and Bork, 2016). For IQtree construction, we utilized buffalo whole-genome mtDNA (CM034296.1) as an outgroup for reference obtained from NCBI.

### Genome-wide selection signatures

We investigated selection signatures in the genomes of Chinese and Bangladesh-Myanmar Gayal by analyzing several genetic parameters and approaches. To assess population differentiation, we employed *F*_ST_ analysis using VCFtools, applying a 50 kb sliding window with a 20 kb step size. We also examined cross-population selection signals using *F*_ST_, θπ-ratio, XP-CLR and XP-EHH methods. For the *F*_ST_and θπ-ratio analyses, we utilized VCFtools with the same sliding window approach (Hudson et al. 1992; Sabeti et al. 2002). We calculated XP-EHH scores for each population in 50 kb windows using the selscan v1.1 tool (Szpiech and Hernandez 2014). Additionally, we computed average XP-CLR with a 50-kb sliding window and a 20-kb step size (Chen et al. 2010). The candidate selection sweep regions were determined by selecting the top 5% of windows identified by at least two of these methods, ensuring a comprehensive and rigorous analysis of potential selection signatures.

### Enrichment of candidate genes under positive selection

We performed functional annotation and gene enrichment analysis to uncover potential biological processes linked to the Gayal population using KEGG Orthology Based Annotation System (KOBAS)(http://kobas.cbi.pku.edu.cn/kobas3) (Bu et al. 2021). Significantly enriched genes within KEGG pathways and GO terms were identified based on a statistical threshold of corrected *P* value < 0.05.

## Supplementary Information

Supplementary Material 1.

## Data Availability

Newly sequenced Gayal genome data were deposited in GenBank (Bio Project accession number: PRJNA1022603).

## Abbreviations

IUCN: International Union for Conservation of Nature and Natural Resources
SNP: Single nucleotide polymorphism
LD: Linkage disequilibrium
NJ tree: Neighbor-joining tree
PCA: Principal component analysis
ML tree: Maximum likelihood tree
*F*_ST_: Fixation index
θπ-ratio: Nucleotide diversity ratio
XP-CLR: Cross population composite likelihood ratio
XP-EHH: Cross-population extended haplotype homozygosity
KEGG: Kyoto Encyclopedia of Genes and Genomes
GO: Gene Ontology
EAMC: Experimental Animal Management Committee
bp: Base pairs
NCBI: National Center for Biotechnology Information
PCR: Polymerase Chain Reaction
MAF: Minor allele frequency
GATK: Genome Analysis Toolkit
MIA: Mapping Iterative Assembler
KOBAS: KEGG Orthology Based Annotation System

## References

[CR1] Alexander DH, Novembre J, Lange KJGR (2009) Fast model-based estimation of ancestry in unrelated individuals. Genome Res 19(9):1655–1664. 10.1101/gr.094052.109 19648217 10.1101/gr.094052.109 PMC2752134

[CR2] Baig M, Mitra B, Qu K, Peng MS, Ahmed I, Miao YW, Zan LS, Zhang YP (2013) Mitochondrial DNA diversity and origin of Bos frontalis. Curr Sci 10:115–120

[CR3] Belenguer G, Mastrogiovanni G, Pacini C, Hall Z, Dowbaj AM, Arnes-Benito R, Sljukic A, Prior N, Kakava S, Bradshaw CR (2022) RNF43/ZNRF3 loss predisposes to hepatocellular-carcinoma by impairing liver regeneration and altering the liver lipid metabolic ground-state. Nat Commun 13(1):334. 10.1038/s41467-021-27923-z 35039505 10.1038/s41467-021-27923-z PMC8764073

[CR4] Bolger AM, Lohse M, Usadel B (2014) Trimmomatic: a flexible trimmer for Illumina sequence data. Bioinformatics 30(15):2114–2120. 10.1093/bioinformatics/btu170 24695404 10.1093/bioinformatics/btu170 PMC4103590

[CR5] Briggs AW, Good JM, Green RE, Krause J, Maricic T, Stenzel U, Lalueza-Fox C, Rudan P, Brajkovic D, Kucan Z, Gusic I, Schmitz R, Doronichev VB, Golovanova LV, de la Rasilla M, Fortea J, Rosas A, Pääbo S (2009) Targeted retrieval and analysis of five Neandertal mtDNA genomes. Science 17:325(5938):318–321. 10.1126/science.1174462 10.1126/science.1174462 19608918

[CR6] Browning SR, Browning BL (2007) Rapid and accurate haplotype phasing and missing-data inference for whole-genome association studies by use of localized haplotype clustering. Am J Hum Genet 81(5):1084–1097. 10.1086/521987 17924348 10.1086/521987 PMC2265661

[CR7] Bu D, Luo H, Huo P, Wang Z, Zhang S, He Z, Wu Y, Zhao L, Liu J, Guo J (2021) KOBAS-i: intelligent prioritization and exploratory visualization of biological functions for gene enrichment analysis. Nucleic Acids Res 49(W1):W317–W325. 10.1093/nar/gkab447 34086934 10.1093/nar/gkab447 PMC8265193

[CR8] Chen H, Patterson N, Reich D (2010) Population differentiation as a test for selective sweeps. Genome Res 20(3):393–402. 10.1101/gr.100545.109 20086244 10.1101/gr.100545.109 PMC2840981

[CR9] Chi J, Fu B, Nie W, Wang J, Graphodatsky A, Yang F (2004) New insights into the karyotypic relationships of Chinese muntjac (Muntiacus reevesi), forest musk deer (Moschus berezovskii) and gayal (Bos frontalis). Cytogenet Genome Res 108(4):310–316. 10.1159/000081520 10.1159/000081520 15627750

[CR10] Danecek P, Auton A, Abecasis G, Albers CA, Banks E, DePristo MA, Handsaker RE, Lunter G, Marth GT, Sherry STJB (2011) The variant call format and VCFtools. Bioinformatics 27(15):2156–2158. 10.1093/bioinformatics/btr330 21653522 10.1093/bioinformatics/btr330 PMC3137218

[CR11] Deng W, Wang L, Ma S, Jin B, He T, Yang Z, Mao H, Wanapat M (2007) Comparison of Gayal (Bos frontalis) and Yunnan Yellow cattle (Bos taurus): rumen function, digestibilities and nitrogen balance during feeding of pelleted lucerne (Medicago sativum). Asian Australas J Anim Sci 20(6):900–907. 10.5713/ajas.2007.900

[CR12] Dorji T, Wangdi J, Shaoliang Y, Chettri N, Wangchuk K (2021) Mithun (*Bos frontalis*): the neglected cattle species and their significance to ethnic communities in the Eastern Himalaya - A review. Anim Biosci 34(11):1727–1738. 10.5713/ab.21.0020 10.5713/ab.21.0020 PMC8563247 33902178

[CR13] Gallagher DJ, Womack JE (1992) Chromosome conservation in the Bovidae. J Hered 83(4):287–298. 10.1093/oxfordjournals.jhered.a111215 1401875 10.1093/oxfordjournals.jhered.a111215

[CR14] Ganapathi M, Friocourt G, Gueguen N, Friederich MW, Le Gac G, Okur V et al (2022) A homozygous splice variant in ATP5PO, disrupts mitochondrial complex V function and causes Leigh syndrome in two unrelated families. J Inherit Metab Dis 45(5):996–1012. 10.1002/jimd.12526 10.1002/jimd.12526 PMC9474623 35621276

[CR15] Gautier M, Klassmann A, Vitalis R (2017) rehh 2.0: a reimplementation of the R package rehh to detect positive selection from haplotype structure. Mol Ecol Resour 17(1):78–90. 10.1111/1755-0998.12634 10.1111/1755-0998.12634 27863062

[CR16] Ge C, Tian Y, Chen T, Wu Y (1996) Studies on the meat feature of gayal (*Bos Frontalis*). Sci Agric Sin 29:75–78

[CR17] Giasuddin M, Huque K, Alam J (2003) Reproductive potentials of gayal (Bos frontalis) under semi-intensive management. Asian Australas J Anim Sci 16(3):331–334. 10.5713/ajas.2003.331

[CR18] Gou X, Wang Y, Yang S, Deng W, Mao H (2010) Genetic diversity and origin of Gayal and cattle in Yunnan revealed by mtDNA control region and SRY gene sequence variation. J Anim Breed Genet 127(2):154–160. 10.1111/j.1439-0388.2009.00807.x 20433524 10.1111/j.1439-0388.2009.00807.x

[CR19] Hammarsjo A, Wang Z, Vaz R, Taylan F, Sedghi M, Girisha KM, Chitayat D, Neethukrishna K, Shannon P, Godoy R, Gowrishankar K, Lindstrand A, Nasiri J, Baktashian M, Newton PT, Guo L, Hofmeister W, Pettersson M, Chagin AS, Nishimura G, Yan L, Matsumoto N, Nordgren A, Miyake N, Grigelioniene G, Ikegawa S (2017) Novel KIAA0753 mutations extend the phenotype of skeletal ciliopathies. Sci Rep 7(1):15585. 10.1038/s41598-017-15442-1 29138412 10.1038/s41598-017-15442-1 PMC5686170

[CR20] He Z, Qu K, Yuan X, San Y, Ma W, Zhang J, Li Z (2009) Appearance characteristics and major behavior of gayal (Bos frontalis) on the conservation ex situ in Pheonix mountains. J Yunnan Agric Univ (Nat. Sci. Ed.) 24(2):25–30

[CR21] Hudson RR, Slatkin M, Maddison WP (1992) Estimation of levels of gene flow from DNA sequence data. Genetics 132(2):583–589. 10.1093/genetics/132.2.583 10.1093/genetics/132.2.583 PMC1205159 1427045

[CR22] Huque K, Rahman M, Jalil M (2001) Study on the growth pattern of gayals (Bos frontalis) and their crossbred calves. Asian Australas J Anim Sci 14(9):1245–1249. 10.5713/ajas.2001.1245

[CR23] Kory N, Wyant GA, Prakash G, uit de Bos J, Bottanelli F, Pacold ME, Chan SH, Lewis CA, Wang T, Keys HR, Guo YE, Sabatini DM (2018) SFXN1 is a mitochondrial serine transporter required for one-carbon metabolism. Science 362(6416):eaat9528. 10.1126/science.aat9528 10.1126/science.aat9528 PMC6300058 30442778

[CR24] Kumar S, Stecher G, Tamura K (2016) MEGA7: Molecular Evolutionary Genetics Analysis Version 7.0 for Bigger Datasets. Mol Biol Evol 33(7):1870–1874. 10.1093/molbev/msw054 27004904 10.1093/molbev/msw054 PMC8210823

[CR25] Lan H, Xiong X, Lin S, Liu A, Shi L (1993) Mitochondrial DNA polymorphism of cattle (Bos taurus) and mithun (Bos frontalis) in Yunnan Province. Acta Genet Sini 20(5):419–425 8161472

[CR26] Letunic I, Bork P (2016) Interactive tree of life (iTOL) v3: an online tool for the display and annotation of phylogenetic and other trees. Nucleic Acids Res 44(W1):W242-W245. 10.1093/nar/gkw290 10.1093/nar/gkw290 PMC4987883 27095192

[CR27] Li H, Handsaker B, Wysoker A, Fennell T, Ruan J, Homer N, Marth G, Abecasis G, Durbin R (2009) The sequence alignment/map format and SAMtools. Bioinformatics 25(16):2078–2079. 10.1093/bioinformatics/btp352 19505943 10.1093/bioinformatics/btp352 PMC2723002

[CR28] Ma G, Chang H, Li S, Chen H, Ji D, Geng R, Chang C, Li Y (2007) Phylogenetic relationships and status quo of colonies for gayal based on analysis of cytochrome b gene partial sequences. J Genet Genomics 34(5):413–419. 10.1016/s1673-8527(07)60045-9 17560527 10.1016/S1673-8527(07)60045-9

[CR29] Mannen H, Kohno M, Nagata Y, Tsuji S, Bradley DG, Yeo JS, Nyamsamba D, Zagdsuren Y, Yokohama M, Nomura K, Amano T (2004) Independent mitochondrial origin and historical genetic differentiation in North Eastern Asian cattle. Mol Phylogenet Evol 32(2):539–544. 10.1016/j.ympev.2004.01.010 15223036 10.1016/j.ympev.2004.01.010

[CR30] McKenna A, Hanna M, Banks E, Sivachenko A, Cibulskis K, Kernytsky A, Garimella K, Altshuler D, Gabriel S, Daly M (2010) The genome analysis toolkit: a MapReduce framework for analyzing next-generation DNA sequencing data. Genome Res 20(9):1297–1303. 10.1101/gr.107524.110 20644199 10.1101/gr.107524.110 PMC2928508

[CR31] Mei C, Wang H, Zhu W, Wang H, Cheng G, Qu K, Guang X, Li A, Zhao C, Yang W (2016) Whole-genome sequencing of the endangered bovine species Gayal (Bos frontalis) provides new insights into its genetic features. Sci Rep 6(1):19787. 10.1038/srep19787 26806430 10.1038/srep19787 PMC4726396

[CR32] Mondal M, Dhali A, Rajkhowa C, Prakash BS (2004) Secretion patterns of growth hormone in growing captive mithuns (*Bos frontalis*). Zoolog Sci 21(11):1125–1129. 10.2108/zsj.21.1125 15572864 10.2108/zsj.21.1125

[CR33] Nguyen LT, Schmidt HA, von Haeseler A, Minh BQ (2015) IQ-TREE: a fast and effective stochastic algorithm for estimating maximum-likelihood phylogenies. Mol Biol Evol 32(1):268–274. 10.1093/molbev/msu300 25371430 10.1093/molbev/msu300 PMC4271533

[CR34] Nyunt M, Win N (2004) Mithan (Bos frontalis) in Myanmar. Rep Soc Res Nativ Livest 21:19–22

[CR35] Patitucci C, Hernandez-Camacho JD, Vimont E, Yde S, Cokelaer T, Chaze T, Giai GQ, Matondo M, Gazi A, Nemazanyy I, Stroud DA, Hock DH, Donnarumma E, Wai T (2023) Mtfp1 ablation enhances mitochondrial respiration and protects against hepatic steatosis. Nat Commun 14(1):8474. 10.1038/s41467-023-44143-9 38123539 10.1038/s41467-023-44143-9 PMC10733382

[CR36] Patrick GJ, Liu H, Alphonse MP, Dikeman DA, Youn C, Otterson JC et al (2021) Epicutaneous Staphylococcus aureus induces IL-36 to enhance IgE production and ensuing allergic disease. J Clin Invest 131(5):e143334. 10.1172/jci143334 10.1172/JCI143334 PMC7919715 33645549

[CR37] Patterson N, Price AL, Reich D (2006) Population structure and eigenanalysis. PLoS Genet 2(12):e190. 10.1371/journal.pgen.0020190 17194218 10.1371/journal.pgen.0020190 PMC1713260

[CR38] Purcell S, Neale B, Todd-Brown K, Thomas L, Ferreira MA, Bender D, Maller J, Sklar P, de Bakker PI, Daly MJ, Sham PC (2007) PLINK: a tool set for whole-genome association and population-based linkage analyses. Am J Hum Genet 81(3):559–575. 10.1086/519795 17701901 10.1086/519795 PMC1950838

[CR39] Qian X, Li X, Shi Z, Bai X, Xia Y, Zheng Y, Xu D, Chen F, You Y, Fang J (2019) KDM3A senses oxygen availability to regulate PGC-1α-mediated mitochondrial biogenesis. Mol Cell 76(6):885–895. 10.1016/j.molcel.2019.09.019 10.1016/j.molcel.2019.09.019 31629659

[CR40] Qu K, He Z, Nie W, Zhang J, Jin X, Yang G, Yuan X, Huang B, Zhang Y, Zan L (2012) Karyotype analysis of mithun (Bos frontalis) and mithun bull x Brahman cow hybrids. Genet Mol Res 11:131–140. 10.4238/2012.january.19.1 22290473 10.4238/2012.January.19.1

[CR41] Sabeti PC, Reich DE, Higgins JM, Levine HZ, Richter DJ, Schaffner SF et al (2002) Detecting recent positive selection in the human genome from haplotype structure. Nature 419(6909):832–837. 10.1038/nature01140 10.1038/nature01140 12397357

[CR42] Sambrook J, Fritsch EF, Maniatis T (1989) Molecular cloning: a laboratory manual. Cold Spring Harbor Laboratory Press, New York

[CR43] Shan XN, Chen YF, Luo LH, Cao XM, Song JZ, Zeng YZ (1980) The karyotype analysis of Gayal. Hereditas (Beijing) 2(5):25–27

[CR44] Stephen J, Vilboux T, Mian L, Kuptanon C, Sinclair CM, Yildirimli D, Maynard DM, Bryant J, Fischer R, Vemulapalli M, Mullikin JC, Huizing M, Gahl WA, Malicdan M, Gunay-Aygun M (2017) Mutations in KIAA0753 cause Joubert syndrome associated with growth hormone deficiency. Hum Genet 136(4):399–408. 10.1007/s00439-017-1765-z 28220259 10.1007/s00439-017-1765-z PMC5395200

[CR45] Szpiech ZA, Hernandez RD (2014) selscan: an efficient multithreaded program to perform EHH-based scans for positive selection. Mol Biol Evol 31(10):2824–2827. 10.1093/molbev/msu211 25015648 10.1093/molbev/msu211 PMC4166924

[CR46] Tanaka K, Takizawa T, Murakoshi H, Dorji T, Nyunt MM, Maeda Y, Yamamoto Y, Namikawa T (2011) Molecular phylogeny and diversity of Myanmar and Bhutan mithun based on mtDNA sequences. Anim Sci J 82(1):52–56. 10.1111/j.1740-0929.2010.00819.x 21269359 10.1111/j.1740-0929.2010.00819.x

[CR47] Uzzaman MR, Bhuiyan MSA, Edea Z, Kim K-S (2014) Semi-domesticated and Irreplaceable genetic resource gayal (Bos frontalis) needs effective genetic conservation in Bangladesh: a review. Asian Australas J Anim Sci 27(9):1368–1372. 10.5713/ajas.2014.14159 25178382 10.5713/ajas.2014.14159 PMC4150205

[CR48] Wang K, Li M, Hakonarson H (2010) ANNOVAR: functional annotation of genetic variants from high-throughput sequencing data. Nucleic Acids Res 38(16):e164–e164. 10.1093/nar/gkq603 20601685 10.1093/nar/gkq603 PMC2938201

[CR49] Wu DD, Ding XD, Wang S, Wojcik JM, Zhang Y, Tokarska M, Li Y, Wang MS, Faruque O, Nielsen R, Zhang Q, Zhang YP (2018) Pervasive introgression facilitated domestication and adaptation in the Bos species complex. Nat Ecol Evol 2(7):1139–1145. 10.1038/s41559-018-0562-y 10.1038/s41559-018-0562-y 29784979

[CR50] Wu Z, Zhang Z, Lei Z, Lei P (2019) CD14: Biology and role in the pathogenesis of disease. Cytokine Growth Factor Rev 48:24–31. 10.1016/j.cytogfr.2019.06.003 31296363 10.1016/j.cytogfr.2019.06.003

[CR51] Xi D, Wu M, Fan Y, Liu Q, Leng J, Gou X, Mao H, Deng W (2012) Polymorphisms of the insulin-like growth factor-binding protein 3 gene (IGFBP3) in gayal (Bos frontalis). Gene 497(1):98–102. 10.1016/j.gene.2012.01.051 22310386 10.1016/j.gene.2012.01.051

[CR52] Yamaji M, Jishage M, Meyer C, Suryawanshi H, Der E, Yamaji M, Garzia A, Morozov P, Manickavel S, McFarland HL, Roeder RG, Hafner M, Tuschl T (2017) DND1 maintains germline stem cells via recruitment of the CCR4-NOT complex to target mRNAs. Nature 543(7646):568–572. 10.1038/nature21690 28297718 10.1038/nature21690 PMC5488729

[CR53] Yuan X, Zheng H, Su Y, Guo P, Zhang X, Zhao Q, Ge W, Li C, Xi Y, Yang X (2019) Drosophila Pif1A is essential for spermatogenesis and is the homolog of human CCDC157, a gene associated with idiopathic NOA. Cell Death Dis 10(2):125. 10.1038/s41419-019-1398-3 30741974 10.1038/s41419-019-1398-3 PMC6370830

[CR54] Zhang Z, Shen X, Gude DR, Wilkinson BM, Justice MJ, Flickinger CJ, Herr JC, Eddy EM, Strauss JR (2009) MEIG1 is essential for spermiogenesis in mice. Proc Natl Acad Sci USA 106(40):17055–17060. 10.1073/pnas.0906414106 19805151 10.1073/pnas.0906414106 PMC2746124

[CR55] Zhang C, Dong SS, Xu JY, He WM, Yang TL (2019) PopLDdecay: a fast and effective tool for linkage disequilibrium decay analysis based on variant call format files. Bioinformatics 35(10):1786–1788. 10.1093/bioinformatics/bty875 30321304 10.1093/bioinformatics/bty875

